# Esophageal groove dysfunction: a cause of ruminal bloat in newborn calves

**DOI:** 10.1186/s12917-018-1573-2

**Published:** 2018-09-10

**Authors:** Tamirat Kaba, Berhanu Abera, Temesgen Kassa

**Affiliations:** 1 College of Veterinary Medicine, Haramaya University, P.O.Box-138, Dire Dawa, Ethiopia; 2 Ethiopian Institutes of Agricultural Research, Holleta Agricultural Research Center, P.O. Box: 2003, Holleta, Ethiopia

**Keywords:** Esophageal groove, Ruminal bloat, Rumenostomy

## Abstract

**Background:**

Esophageal groove dysfunction is one of the major causes of ruminal bloat. This condition is fatal in new born calves if it is not treated early. In healthy, suckling calves, milk should bypass the forestomach (rumen and reticulum) and enter into the abomasum where enzymatic digestion of milk proteins takes place. However, failure of the esophageal groove allows milk to enter into the forestomach, which results in the production of excess gases by microbial fermentation. Consequently, this increase in abdominal distention particularly on the left side in ruminants is an imminent manifestation of excess gases in the foresomach.

**Case presentation:**

A 10-day-old crossbred male calf presented with a distended left abdomen and manifesting dyspnea at a dairy farm. The calf was weak, reluctant to move, and had visibly congested mucus membranes. Regarding the calf’s feeding, milk was the only thing ingested and the calf had not started on dry feeds (hay, concentrates, and roughages). According to the herdsman of the farm, the calf had a mild-to-moderate form of bloat and 3–5 h after milk feeding the bloat would disappear spontaneously. During bloat, an increase in pulse rate, respiratory rate (tachypnea), and shallow breathing was noted. Physical examination revealed severe distention of the left side of the abdomen, and on percussion, accumulation of gases mixed with fluid in the left abdomen was detected. An attempt was made to release gases from forestomach by introducing a stomach tube with oral antibiotics; however, the case was not resolved. The calf suffered from frequent recurrence of bloat after every milk feed, and in response to the refractory outcome to conventional treatment, a rumenostomy was indicated and a better treatment response was achieved. In addition, IV fluid and other supportive therapy were provided while milk was withheld. However, considering the fact that milk is a natural feed that should not be taken away from every calf at this age, we had to encourage calf to consume milk as it would not result in bloat as far as rumen fistula is being created. Furthermore, encouraging calves to consume starter feed (fresh grasses and hay) earlier than usual recommended period whilst decreasing milk intake would hasten the rumen function.

**Conclusions:**

Cases like this are successfully managed by a rumenostomy when conventional options fail.

## Background

Calf mortality has been an important problem in the dairy industry for more than a hundred years, and the causes of death are multifactorial, from environmental and infectious agents, to host phenotype. Although the knowledge about neonatal diseases in calves has increased in recent years, the mortality rate is still rather high [1]. One of the major reasons for high mortality rate among neonatal calves is ruminal bloat. Ruminal bloat occurs when gas produced during fermentation builds up in the rumen and is unable to escape. It is usually a secondary problem in newborn calves. Ruminal bloat can become life threatening within a few hours and often requires medical attention [2]. The most common cause of ruminal bloat in calves that solely consume milk, is failure of esophageal groove closure [3]. For the first two weeks after birth, a calf is monogastric, a simple stomached animal, using only the abomasum to digest the milk or milk replacer. When the calf suckles milk, milk bypasses the rumen and reticulum to enter into the abomasum, where digestion and absorption takes place. Milk entering into the rumen and reticulum is both wasteful and dangerous to the newborn calf; hence the importance of the esophageal groove in diverting milk from the esophagus into the abomasum [4].

The physiology of esophageal groove closure was studied by many scholars. For instance, in 1826**,** Tiedeman and Gmelin were the first workers to report that milk passed directly to the abomasum in young lambs and calves [5]. Since then, a couple of physiologists had investigated what triggers the closure of the esophageal groove and have come up with very diverging opinions. Colin [6] concluded that the “esophageal groove closed when boli was swallowed during rumination and that this was the main route for passage of solid matter from the rumen to the omasum and abomasum”. Schalk and his colleague [7], and a non-peer reviewed compilation Costello, [8] and Wise [9] suggested that if calves suckled milk from a rubber nipple it usually passed into the abomasum, while it often passed to the rumen if it was drunk from a bucket. They concluded that the closure of the esophageal groove is trigged when the calf directly suckled the milk from a dam. Larry [10] stated that the esophageal groove closure depends upon the liquid ingested which stimulates the nerve receptors in the mouth. Other studies suggest that esophageal groove closure and dilatation of the omaso-abomasal canal is initiated by the stimulation of the vagus nerve through contact with sensory receptors in the oral cavity and pharyngeal area [11]. Gradually (after a few weeks of weaning), this response fades so that the groove is no longer functional. Dysfunction of the esophageal groove results in leakage of fluid into the forestomach. Spillage from the esophageal groove may result from either a complete failure of groove closure or sequential opening and closure during drinking. According to Gentile [12] pathological conditions (diarrhea, phlebitis of jugular vein, cough, otitis and anorexia), irregular feeding (irregular feeding times, forceful feeding, bucket feeding of milk, abnormal milk temperature) and stress factors (long distance transportation) are some of the causes of esophageal groove dysfunction. However, many studies [3, 8, 11] indicate that esophageal groove dysfunction is unusual in calves that suckle directly from the dam. This report presents a single clinical case of ruminal bloat associated with a putative esophageal groove dysfunction in a 10-day-old calf. We believe that the therapeutic intervention made in the field was a better management approach in respect to the area’s lack of facilities to conduct a laboratory investigation.

## Case presentation

**Fig. 1:**
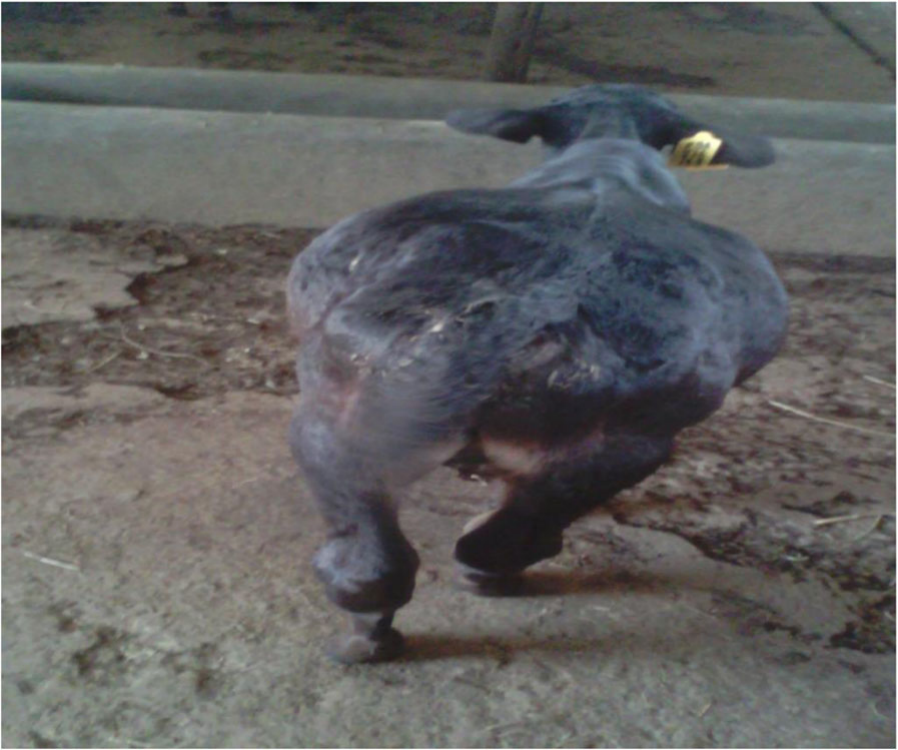
A Photo showing ruminal bloat in 10 days old crossbred calf

A 10-day-old male crossbred (Frisian x local indigenous) calf presented with a severely distended abdomen (Fig. 1). Due to the distention the paralumbar fossa, especially on the left, was not visible. The calf was reluctant to suckle from the dam, unable to walk, exhibited rapid and shallow breathing, and had visibly congested mucus membranes. Percussion of the left abdomen revealed a drum-like gaseous sound. On auscultation of the left abdomen, a dull fluid sound was detected. The anamnesis indicated that the calf had been dribbling urine continuously, unable to defecate, or had irregularly voided very little, hard, and pasty feces. General physical examination revealed no esophageal obstruction, but the calf was weak and with an abnormal gait. The calf was suckling its dam twice in a 12 h interval (at morning and evening) and had not started feeding the hay/roughage/concentrate or the calf starter at the moment. The physiological parameters of the calf were as follows: Rectal temperature = 39.8 degree Centigrade (°C), Pulse =175 beats/minute, Respiration =60 breaths/minute.

### Differential diagnosis

Abomasal bloat and choke.

### Treatment approach

In order to release trapped gases and check the patency of the esophagus, a flexible stomach tube coated with mineral oil was inserted into the esophagus, and advanced down into the rumen. A fermented watery-like fluid accompanied by some clots of milk and gases was released from the rumen through the stomach tube. Procaine penicillin (Pen Aqueous; Zoetis Canada), 10 ml (ml), 10,000 international unit per milliliter (Iu/ml of solution) mixed with 0.25Liter (L) of mineral oil was administered orally for 3 days, while milk was withheld to reduce the microbial burden and coalescence of gas. An isotonic solution containing 0.9% Sodium Chloride (Jiangsu HFQ Bio-Technology Co., Ltd), 8.4% Sodium Bicarbonate (Vet One, Nova-Tech, Grand Island, USA) and 5% Dextrose in water 1000 ml injection (Addis Pharmaceutical factory) was administered intravenously (IV) at a rate of 100 ml/kilogram (kg) over 3–5 h for 2 days. Before administration of IV fluid, the calf was sedated using Xylazine hydrochloride, 20 mg/ml (xylazine® immunological LTD, Hyderabad, India) intramuscularly (IM). This was administered during every fluid therapy, and the calf was tied up with rope in a lateral recumbent position. The hair around the jugular groove of the neck was clipped and the area was cleaned and disinfected using diluted 70% Ethanol (Addis Pharmaceutical factory). The superficial jugular vein was catheterized using 20 Gage, 0.8 in. butterfly catheter (Unolok, Hindustan syringe, Medical device LTD Faridabad, India) and secured with adhesive tape around the neck.

### Response to treatment

After 3 days of treatment, bloat reoccurred. Treatment was initiated a second time by giving antibiotic pen strep (Pen & Strep^@^_,_ 100 ml, York Vet, USA): 5 ml, (IM), every 24 h (q24hrs) for 2 days while the calf had been fastening. Additional supportive therapy of 40% glucose (100 ml/kg/day IV), isotonic saline solution (10 ml/kg/hrs IV) and a multivitamin (Multivitamin injection 100 ml, Norbrook Laboratories Limited, Ireland), was administered 10 ml IM once at a time (Stat.) during the time that the milk was withheld. After 2 days of treatment, the calf was allowed to suckle milk from the dam; however, the calf exhibited bloat again 5 h after milk consumption.

### Rumenostomy

**Fig. 2:**
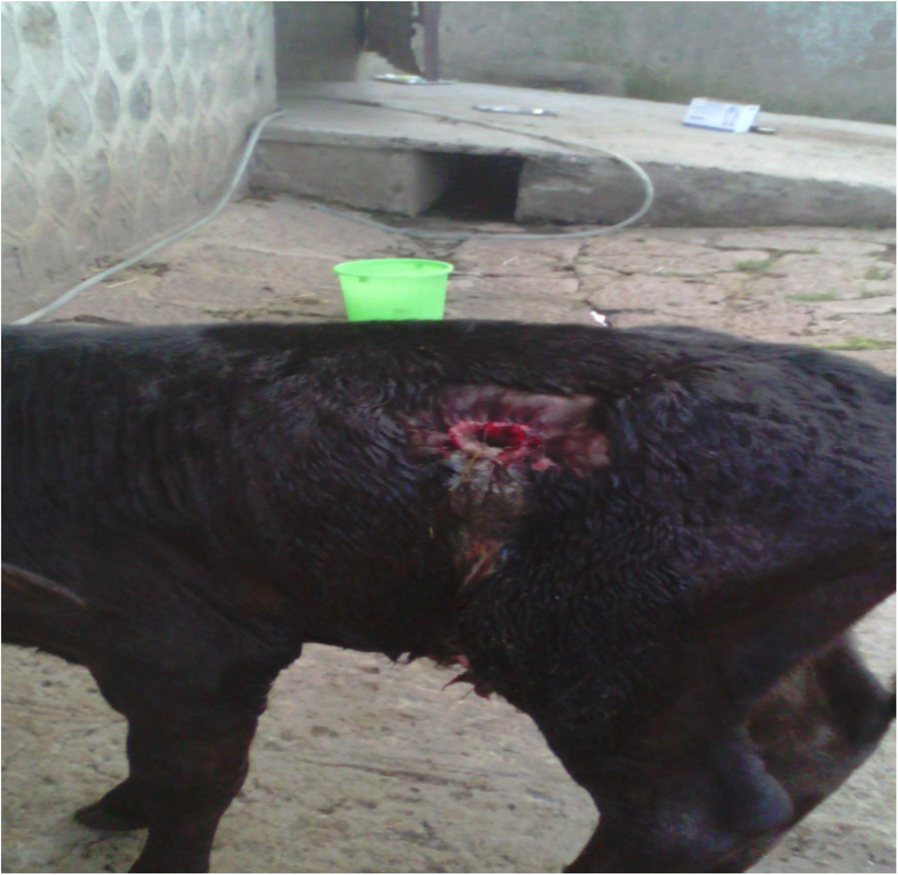
A photo showing a calf with fistulated rumen (taken right after surgery)

Ruminal fistulation (rumenostomy**)** was conducted to prevent recurrence according to a procedure described by Turner and Mcilwraith [13]. Before the surgical procedure milk was withheld from the calf overnight while IV fluids and glucose were administered at the dose rate explained above. The left paralumbar fossa was prepared by shaving the hair and washing skin aseptically using 7.5% povidone-iodine surgical scrub (Povidone-iodine cleansing solution, Wockhard LTD, Mumbai, India) while the calf was standing. A circular area of 6 cm (cm) in diameter just below the transverse process of the lumbar vertebrae was marked and infiltrated with local anesthetic, 2% Lidocaine (Zoetis Canada, Kirkland,Quebec), at the concentration of 20 mg per milliliter (mg/ml). Approximately a 2 cm diameter circular incision was made to remove the skin. After skin removal the abdominal muscles were dissected bluntly to expose the rumen. The rumen was grasped using sponge forceps and pulled to the exterior. The rumen wall was then tacked to the edge of the skin by four horizontal mattress sutures at “quarter hour” positions (12, 3, 6 and 9 o’clock). These sutures acted as stay sutures using a non-absorbable suture (Sofsilk™ 6–0 Black, Medtronic, USA). The rumen wall was incised carefully at one half centimeter from the wound margin/apposing skin. As the contents of the rumen came out during the procedure, we observed a high amount of milk that had entered into the rumen (Fig. 2).

### Post Rumenostomy management

The calf was separated from the herd for 10 days to maintain close observation. Since rumenostomy is considered a clean contaminated surgery, we had to give parenteral antibiotic, penstrep, (Penstrep-400, Metaalweg, 85,804 CG Venray, Netherlands) 5 ml for 4 days, q24hrs IM to reduce the risks of peritonitis. A dexamethasone injection at 2 mg/ml (Sparhawk laboratories Inc., Lemexa, KS66215, USA) was given every eight hours (q8hrs) IM, and also served as an anti-inflammatory agent. Moreover, Deltamethrin 1% (*w*/*v*) pour-on ready-for-use formulation (Appropriate Applications Ltd., USA) at a dose rate of 10 ml per 100 kg body weight was used to prevent insect infestation and miyiasis. The surgical wound was examined and monitored every day until closure for any complications such as wound dehiscence or rumen attachment to the skin. Rumen contents leaking out onto the flank area and outer surgical site were cleaned by using antiseptic solution (Chlorhexidine) and clean towels. The rumen was repeatedly flushed through the fistula with 0.5–1 l of warm tap water adjusted to the calf’s body temperature. This flushing helped to prevent desiccation and was used for buffering purpose. The calf was allowed to suckle milk from its dam twice a day during the follow-up period. After 10 days post-operation, the calf was provided with some hay and fresh grasses to stimulate rumen function. Bloat resolved by the time the calf started solid feeds and the wound was closed surgically just after a week of feeding grasses and hay.

### Response to Rumenostomy

The calf was followed for 6 months after the procedure. Shortly after wound closure, the amount of milk that the calf was getting was reduced to encourage the intake of hay and grasses. During this time, bloat did not occur as it had been observed prior to surgery. Although ruminal contents spilled onto the flank post-operatively, this did not appear to upset the calf, and its general condition improved gradually. We recommended that the owner reintroduce the calf with the existing herd 6 months after the surgery, and advised the owner to inform us of any observable complications. We promised the owner that we would visit the calf at one year; however, the owner had sold the calf at 9 months of age to a beef farmer in another area of the country.

## Discussion and conclusion

The occurrence of bloat in calves that have started on a hay/grass/concentrate or a calf starter is not a new phenomenon, however, in newborn calves that are only suckling milk, it is unusual [14]. According to some studies, [3, 12] ruminal bloat is a secondary consequence to esophageal groove dysfunction in calves at this age. Esophageal groove dysfunction is the major cause of ruminal bloat in newborn calves that are directly suckling its dam, or could be the result of overfeeding concentrate feed [15]. The later cause of ruminal bloat doesn’t seem to be appearing a factor for this case because the calf wasn’t turned onto concentrate feed at the moment, and was suckling only the milk from its dam. We were confronted with a paradox justification because in some studies [3, 4, 8, 12] esophageal groove dysfunction in calves directly suckling their dam is not common, but failure of the esophageal groove can occur when calves drink cold milk, are tube fed, or fed from a bucket [16]. In normally functioning esophageal grooves, milk should bypass the rumen and reticulum [17]. The presence of milk in the rumen was confirmed during surgery, when the ruminal contents were observed and a high quantity of fermented milk and milk clots were noted. Abomasal bloat can be a differential diagnosis for this case, however, there were certain things that set ruminal bloat apart from abomasal bloat. For instance, in ruminal bloat abdominal distention is higher on the left side [3], which was seen in this case. In addition, during ruminal bloat rumen contents can easily be released out with the help of a stomach tube whereas, in abdominal bloat it is difficult to introduce a stomach tube into the abomasum and flush its contents out while the animal is in a standing position [2]. When we manipulated the stomach tube while the calf was standing, we were confident that the content was coming out from the forestomach. In the case of choke, bloat must accompany drooling of saliva [3] and recurrences should not have occurred after checking the patency of esophagus using the stomach tube.

The largest problem that we have failed to demonstrate was the underlying cause or factor for esophageal groove failure. Gentile [12] reported that pathological conditions (diarrhea, otitis, phlebitis, vagus nerve problem, etc.), inadequate feeding technique (irregular feeding time, bucket feeding, very cold milk feeding etc) and stress are some of the causes of esophageal groove dysfunction. In our investigation, the calf wasn’t exhibiting diarrhea or any other gross pathological conditions except abdominal distention. Furthermore, we also investigated the feeding technique of the calf and realized that suckling was the only feeding technique and it was regular, twice everyday (12 h interval of milk feed per day).

Bloat in older animals is associated with grazing legumes in legume-dominant pastures, feeding high-grain diets, and impaired eructation processes [18]. Despite the primary cause of bloat being multifactorial, it’s clear that the esophageal groove is not functional in those animals as it regresses when they start solid feeds [19]. Hence, “esophageal groove dysfunction” cannot be an ideal term to use to describe bloat in older animals. Apart from other treatment protocols, several scholars [20–22] suggest that rumenostomy is a therapeutic option for animals with recurrent or non-resolving bloat in young or older animals. Amanda et al. 2015 [23] mentioned that of 42 rumenostomy treated cases, 20 cases were indicated for bloat. According to the authors, half of the calves were followed for long periods in the herd and they had better health conditions until they were culled. While the primary associated factor of esophageal groove failure is unclear, the presence of milk clot and fermented fluid in high amounts in the rumen at an early age suggests a malfunction in the normal physiology of the esophageal groove.

To the best of our knowledge, case like this has never been reported so far in naturally suckling calves. As treatment intervention, withholding milk whilst giving IV fluid would give temporary relief. However, considering the fact that milk is a natural feed that should not be taken away from every calf at this age, we rather encourage calves to consume milk as it would not results in bloat as far as rumen fistula is being created. Furthermore, encouraging calves to consume starter feed (fresh grasses and hay) earlier than usual recommended period whilst decreasing milk intake would hasten the rumen function in those calves. Therefore we concluded that esophageal groove dysfunction should be suspected when severe and recurrent bloat occurs in calves that consume only milk by suckling. Nevertheless, since we did not investigate the underlying cause, detailed study on the primary causes of esophageal groove dysfunction in young calves should be encouraged. We also found that rumenostomy is a better management option over conservative approaches in similar clinical cases. Despite rumenostomy considered a better option, it degrades the appearance and the value of the animal, and we suggest additional studies on alternative treatment methods.

## Abbreviations

Im: Intramuscular
Iu: International unit
Iv: Intravenous
q24hrs: every 24 hours
q8hrs: every 8 hours
Stat.: Once at a time
*W*/*V*: weight per volume
